# Breed-Specific Magnetic Resonance Imaging Characteristics of Necrotizing Encephalitis in Dogs

**DOI:** 10.3389/fvets.2017.00203

**Published:** 2017-12-04

**Authors:** Thomas Flegel

**Affiliations:** ^1^Department of Small Animal Medicine, University of Leipzig, Leipzig, Germany

**Keywords:** necrotizing encephalitis, necrotizing leucoencephalitis, necrotizing meningoencephalitis, magnetic resonance imaging, breed specific magnetic resonance imaging

## Abstract

Diagnosing necrotizing encephalitis, with its subcategories of necrotizing leukoencephalitis and necrotizing meningoencephalitis, based on magnetic resonance imaging alone can be challenging. However, there are breed-specific imaging characteristics in both subcategories that allow establishing a clinical diagnosis with a relatively high degree of certainty. Typical breed specific imaging features, such as lesion distribution, signal intensity, contrast enhancement, and gross changes of brain structure (midline shift, ventriculomegaly, and brain herniation) are summarized here, using current literature, for the most commonly affected canine breeds: Yorkshire Terrier, French Bulldog, Pug, and Chihuahua.

## Introduction

Necrotizing encephalitis (NE) includes several breed specific inflammatory brain diseases within the group of meningoencephalitis of unknown etiology/etiology (MUA/MUE). Several synonyms exist for MUA (non-infectious encephalitis, immune mediated encephalitis) reflecting the fact that even though these diseases have been known for decades, the underlying pathology is still not completely understood and achieving a definite diagnosis intra vitam is usually difficult. Based on histopathological features MUA can be subdivided into granulomatous meningoencephalomyelitis (GME) and NE, with the latter comprising Necrotizing Leukoencephalitis (NLE) and necrotizing meningoencephalitis (NME). These subtypes of NE are seen in specific breeds with NLE affecting Yorkshire Terriers (1–9) and French Bulldogs (10, 11), whereas NME affects Pugs (12–21), Maltese (22–24), Chihuahuas (24–33), Pekingese (25, 34), Shih Tzus (24, 25, 35), West Highland White Terriers (36), Papillons (23, 35), Coton de Tulears (35), and Brussels Griffons (35).

The often applied practice of using the rather unspecific term of MUA instead of the exact subclassification (GME, NME, or NLE) in imaging diagnostics is due to the fact, that differentiation based on imaging alone can be difficult. This situation is further complicated by the fact that magnetic resonance imaging (MRI) may generally have only moderate sensitivity in detecting inflammatory intracranial pathology. Abnormalities in brain MRI were found in 76% of dogs with inflammatory cerebrospinal fluid (CSF) (37). Therefore, imaging findings should be interpreted under consideration of the CSF analysis results. However, normal CSF was found in 28.6% of dogs with histologically confirmed NLE and in 14.3% of Pugs with histologically confirmed NME (25). Similarly, a normal CSF nucleated cell count was reported in 12.5% of dogs with NE (38). Therefore, a definite diagnosis of encephalitis may often require histological confirmation based on a brain biopsy (31).

Nevertheless, common MRI features for NME and NLE that allow diagnosing these diseases with a relatively high degree of certainty are known. The typical breed specific MRI characteristics of both diseases in the most frequently affected breeds will be presented. All images provided for illustration are obtained from dogs with histologically confirmed diagnoses. The images can illustrate only some features of the specific disease in a given breed. Illustration of all possible MRI characteristics is beyond the scope of this publication.

## NLE in Yorkshire Terriers

Uni- or bilateral asymmetrical lesions are seen in the telencephalon and diencephalon. The brainstem is often less severely affected. The cerebellum and spinal cord are usually unaffected on magnetic resonance (MR) images even though in rare cases histopathological examination also shows inflammatory foci here (6, 7). Lesion distribution based on MRI has been described in 27 Yorkshire Terrier cases: forebrain (*n* = 25), thalamus (*n* = 10), midbrain (*n* = 8), and caudal brainstem (*n* = 10; 9). Additional syringohydromyelia was seen in 7/27 (26%) dogs. Lesions were multifocal in about 78% of cases. However, diagnosis in these Yorkshire Terriers was described as meningoencephalitis of unknown origin and not as NLE. Therefore, it cannot be ruled out that some of these dogs were affected by other types of encephalitis.

**Figure 1:**
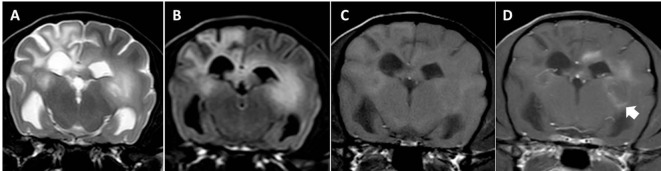
Transverse magnetic resonance images (3 T) of a Yorkshire Terrier (2 years, female) with necrotizing leukoencephalitis. T2 **(A)** and fluid-attenuated inversion recovery **(B)** hyperintense finger-like lesions affecting the subcortical white matter, the corona radiata, and the diencephalon. Right-sided ventricular enlargement, focal widening of sulci, and mild midline shift to the right are most likely secondary to white matter loss in the right prosencephalon. Contrast enhancing lesions as on the left side [**(C)** T1-weighted native; **(D)** T1 weighted after contrast injection] and non-contrast enhancing lesions (right side) may coexist in one patient, which most likely reflects different stages of the disease. Enhancement may be patchy or it may be seen around necrotic areas (ring-like, arrow head). Meningeal contrast enhancement is deficient.

**Figure 2:**
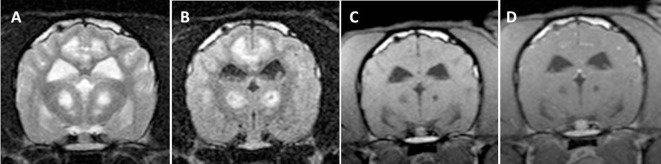
Transverse magnetic resonance images (0.5 T) of a Yorkshire Terrier (3 years, male) with subacute necrotizing encephalopathy. Bilateral symmetric hyperintensities in the cingulate gyrus and in the thalamus on T2-weighted **(A)** and fluid-attenuated inversion recovery **(B)** images. These lesions have a hypointense center on T1-weighted **(C)** images and they do not enhance after a contrast injection **(D)**.

Telencephalic lesions usually affect the periventricular and subcortical white matter, often leaving the overlying cortical gray matter untouched (2, 5). The shape of the lesion may vary from finger-like, following the shape of subcortical white matter, to round-shaped in deep-seated lesions (Figure 1). Therefore, asymmetric thalamic lesions are usually round-shaped and well demarcated. They need to be differentiated from bilateral symmetric round lesions in the telencephalon (including basal nuclei), mid-thalamus and brainstem seen in subacute necrotizing encephalopathy (SNE) of Yorkshire Terriers. Signal intensity on T2-weighted and fluid-attenuated inversion recovery (FLAIR) sequences are similar in both NLE and SNE, but symmetry of lesions should clearly point toward SNE [(39, 40); Figure 2].

Lesions in NLE are usually hyperintense on T2-weighted and FLAIR images and hypointense to isointense on T1-weighted sequences. These signal intensity characteristics may represent multiple areas of cystic necrosis (41). The secondary white matter edema beyond the boundaries of the primary inflammatory lesions is rather mild. On positron emission tomography (PET) imaging, areas of necrosis and cavitation correspond to areas of glucose hypometabolism (30, 42).

In few cases (12%), lesions might be hyperintense on T1 and hypo- or hyperintense on T2-weighted sequences, which most likely correspond to areas of intraparenchymal hemorrhage. The latter could be verified by identification of an intralesional signal void in a T2* sequence. However, studies confirming this assumption in Yorkshire Terriers with NLE are deficient. Midline shift and ventriculomegaly are seen in 50 and 36% of dogs, respectively (25). Both are less likely to be caused by a mass effect of the inflammatory foci, but by a loss of white matter on the more severely affected hemisphere (3, 5). Brain herniation is not a feature of NLE in Yorkshire Terriers.

Lesion enhancement after a contrast injection is usually mild to moderate (5, 7, 25). It can be patchy and inhomogeneous within parenchymal lesions (87%) or ring-like in the periphery of a necrotic lesion corresponding to perinecrotic inflammation (7, 25). The degree of contrast enhancement appears to be related to the degree of lymphohistiocytic inflammation on histological examination (7). Lesions with moderate enhancement and those without enhancement may coexist in one patient (7). The first may reflect active inflammation whereas areas without enhancement may be reflecting burnt out lesions and therefore indicating a more chronic stage of the same disease (5, 7). Meningeal enhancement is lacking in NLE (25). A few Yorkshire Terriers with NLE (7%) may not show any contrast enhancement at all (6, 25).

In addition to NLE, Yorkshire Terriers can also be affected by GME. It may even be seen as a combination of NLE, NME, and GME in the same dog, as described in two cases (43). One displayed typical MRI features of NLE in the prosencephalon, whereas the other had a lesion compatible with GME. Histopathological examination, however, revealed characteristics of NLE, NME, and GME in both dogs.

## NLE in French Bulldogs

**Figure 3:**
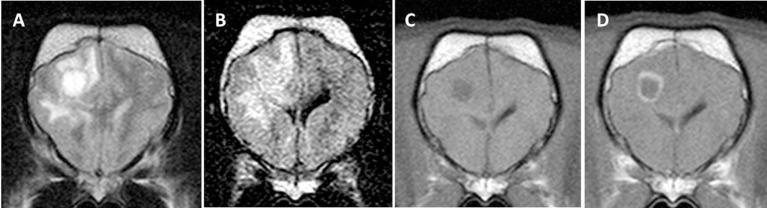
Transverse magnetic resonance images (0.5 T) of a French Bulldog (2 years, female) with necrotizing leukoencephalitis. T2 **(A)** and fluid-attenuated inversion recovery (FLAIR) **(B)** hyperintense round-shaped lesion accompanied by secondary white matter edema in the right frontal lobe. The T2 signal of the round center is partially suppressed on the FLAIR sequence indicating a liquid-like texture being consistent with necrosis. There is a ring-like contrast enhancement around the necrotic center after contrast injection [**(C)** T1-weighted native; **(D)** T1 weighted after contrast injection].

Reports on specific MRI features of NLE in French Bulldogs are limited to three publications regarding four dogs with histologically confirmed disease, with one scan being performed post mortem (10, 11, 25). Lesions may be uni- or multifocal and can be round-shaped and clearly delineated or they may be diffuse. They are T2 and FLAIR hyperintense and can be found in the telencephalon, diencephalon and brainstem, whereas the cerebellum seems to be unaffected. These lesions are iso- to hypointense on T1-weighted images. They may represent necrotic areas in a similar way (as described earlier) for Yorkshire Terriers. However, the primary inflammatory focus is frequently masked by a secondary white matter edema and therefore it may be indistinguishable from secondary edema without a contrast injection. The primary inflammatory focus can often be identified by a mild to moderate peripheral or even ring-like enhancement on T1-weighted images after a contrast injection (Figure 3). More diffuse lesions may commonly show a varying degree of contrast enhancement ranging from a moderate to strong uniform pattern. However, lesions without contrast enhancement can also be seen in the same patient. Meningeal involvement is minimal and therefore it cannot be identified on MRI. Accordingly, the nucleated cell count in CSF is usually only mildly elevated (25). Midline shift is not commonly seen or is only mild. In general, lesions seem to be less severe in French Bulldogs than in Yorkshire Terriers.

One report described inflammatory changes affecting both the optic nerves and retina on histopathology (in addition to those found in the forebrain), which went unnoticed on a post mortem MR image (11).

## NME in Pugs

The majority of Pugs with NME (94%) have multifocal or diffuse, asymmetrical prosencephalic lesions with at least one lesion being located in the telencephalon (21). Lesions are more common in the middle and caudal prosencephalon, being most severe in occipital and parietal lobes, tapering off to rostral with the frontal lobes being less frequently affected (20, 21). About one-third of dogs have additional diencephalic lesions (21).

Brainstem (17%) and cerebellar (22%) involvement are much less common and lesions here are less severe than those in the prosencephalon (21, 25). However, lesion distributions may reflect the limited sensitivity of MRI to detect inflammatory lesions, especially if those are relatively mild, since it is known from a pathological study that 40% of Pugs with NME also have cerebellar lesions (19). In addition, one Pug with NME has been reported with a single brainstem lesion (21). In general, lesion burden in NME can be more dramatic for Pugs compared with other types of NE. However, no correlation could be demonstrated so far between MRI visible lesion severity and prognosis (21).

**Figure 4:**
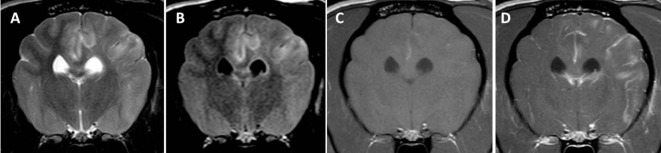
Transverse magnetic resonance images (3 T) of a Pug (3 years, male) with necrotizing meningoencephalitis. Diffuse T2 **(A)** and fluid-attenuated inversion recovery **(B)** hyperintensities predominantly on the left in the prosencephalon resulting in the complete loss of cortical gray and white matter distinction. There is a patchy parenchymal and a leptomeningeal enhancement after a contrast injection [**(C)** T1-weighted native; **(D)** T1 weighted after contrast injection]. The midline is slightly shifted to the right side caused by the expansion of the prosencephalon on the left-hand side.

Lesions being hyperintense on T2-weighted and FLAIR images and mildly hypo- to isointense on T1 images may dominate in the cerebral white matter (about 30% of lesions) or they may more commonly involve gray and white matter to a similar extent. White and gray matter distinction is often missing altogether and corresponds to anatomical disruptions of those structures on histopathological examination (Figure 4). Focal, well-demarcated hyperintensities on T2-weighted images, which follow the white-gray matter boundaries, correlate histologically with white matter edema rather than with areas of macroscopic necrosis of brain parenchyma. Therefore, the latter is not a typical feature of NME in Pugs. Hyperintensity on T2-weighted and FLAIR images in the hippocampus and piriform lobe is mainly consistent with excitotoxic edema caused by long-lasting seizure activity, whereas similar imaging features in other forebrain regions correspond to areas of inflammation or microscopic liquefaction on histopathology (25).

Lesion enhancement after a contrast injection is varying but is usually mild to moderate with an inhomogeneous and patchy intraparenchymal pattern. Meningeal enhancement is seen in slightly more than half of the dogs involving the dura mater and more commonly the leptomeninges (21, 25). The enhancement pattern, corresponding to inflammatory infiltrates found histopathologically in sulci, explains why Pugs are more likely to experience elevated nucleated cell counts on CSF analysis than dogs with NLE (25) and why mean nucleated cell counts are higher in Pugs (250 cell/μl; SD: 172) than in Yorkshire Terriers (19 cells/μl; SD: 21) (25). Between 22 and 43% of Pugs may not show any contrast enhancement at all (21, 25). However, lack of contrast enhancement does not rule out meningitis or encephalitis (37, 44).

**Figure 5:**
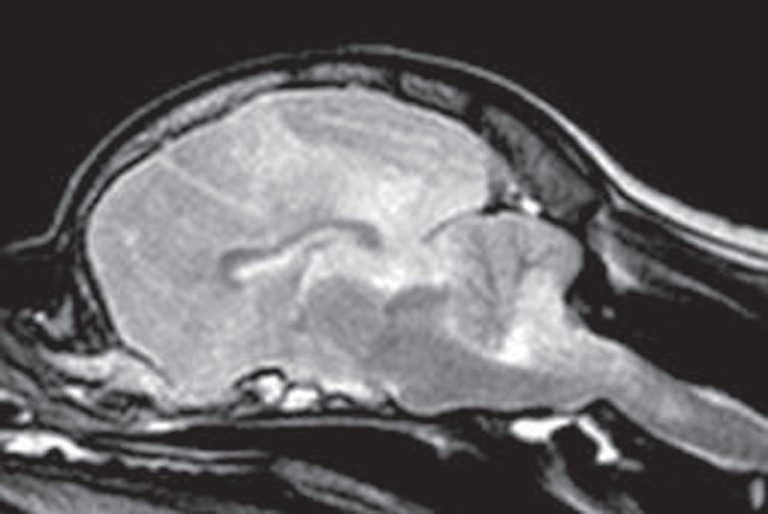
Sagittal T2-weighted magnetic resonance image (0.5 T) of a Pug (9 months, female) with necrotizing meningoencephalitis (NME). Caudal transtentorial herniation of the occipital lobes and foramen magnum herniation of the cerebellum caused by significant swelling of the prosencephalon. This may explain the sudden death of some Pugs with NME.

Midline shift away from the more severely affected brain hemisphere is seen in 25–61% of dogs (21, 25). Different types of brain herniation were detected in about one-third of cases with a caudal transtentorial herniation being most common [(20, 21, 25); Figure 5]. A foramen magnum herniation of the cerebellum is seen in up to 12% of dogs and may contribute to the sudden death observed in some cases (25). Uni- or bilateral ventricular dilation can be identified in about one-third of dogs, with some experiencing signs of intraventricular hypertension such as periventricular edema and dilatation of the olfactory recess. Ventricular dilatation and/or asymmetry may be attributed to the space-occupying effect of inflammatory foci and secondary edema. However, one should keep in mind that it might be just an incidental finding in some cases since ventricular asymmetry can be seen in up to 38% of normal dogs (45).

## NME in Chihuahuas

**Figure 6:**
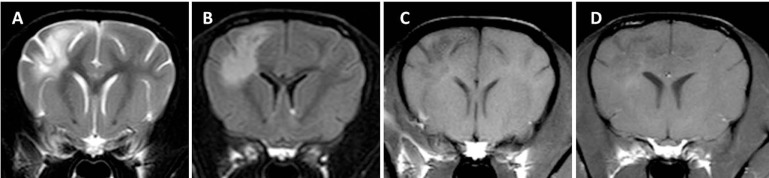
Transverse magnetic resonance images (3 T) of a Chihuahua (11 months, female) with necrotizing meningoencephalitis. T2 **(A)** and fluid-attenuated inversion recovery **(B)** hyperintense finger-like lesions affecting the subcortical white and gray matter in the right prosencephalon. These characteristics may resemble those of Yorkshire Terriers with necrotizing leucoencephalitis since there might be a thin rim of intact cortical gray matter. However, involvement of gray matter can be visualized in most cases. The lesion is hypointense on T1-weighted images **(C)** and is only mildly enhanced in the periphery of the lesion **(D)**.

**Figure 7:**
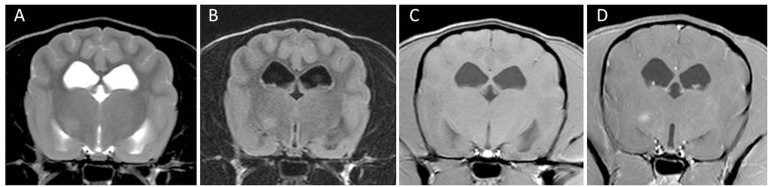
Transverse magnetic resonance images (3 T) of a Chihuahua (5 years, female) with necrotizing meningoencephalitis. Single T2 **(A)** and fluid-attenuated inversion recovery **(B)** hyperintense round-shaped lesion in the right diencephalon. There is a small uniform contrast enhancement in the center of the lesion on the post-contrast T1-weighted image **(D)** compared to the pre-contrast T1-weighted image **(C)**.

Magnetic resonance imaging of two dogs brains demonstrated multifocal loss of cortical gray/white matter demarcation and marked thinning and collapse of the right parietal, temporal, and occipital cortices (29). In contrast to NME in Pugs, a small rim of overlying cortical gray matter might be spared resulting in an MRI that may resemble NLE in Yorkshire Terriers (Figure 6). Involvement of deep white matter such as the capsula interna and diencephalon is also described [(25, 30, 32); Figure 7]. The lesions were hyperintense on T2-weighted images, with T2-weighted hypointensity and slight post-contrast enhancement (29). Brainstem and cerebellum seem to be largely unaffected (29). Lesions correspond to intensely cellular, non-suppurative meningoencephalitis, often with cystic necrosis in subcortical white matter (29). On fluorine-18 fluorodeoxyglucose-PET scans the lesions correspond to areas of hypometabolism (30). CSF analysis can be normal in some dogs and hinders establishing a definite intra vitam diagnosis (29).

In the case of Chihuahuas, the situation is further complicated by the fact that GME can also be seen in this breed and therefore has to be differentiated from NME (25, 46).

## NE in Other Canine Breeds

Imaging characteristics of the four breeds described here are summarized in Table 1. For the other breeds, having been reported to be affected by either NLE or NME, none or only single case reports of MRI characteristics are available and therefore generalizations do not seem feasible.

**Table 1:** Summary of typical imaging characteristics of necrotizing encephalitis in Yorkshire Terriers, French bulldogs, Pug dogs, and Chihuahuas.

Imaging characteristics	Yorkshire Terrier	French bulldog	Pug	Chihuahua
Type of encephalitis	NLE	NLE	NME	NME

Lesion distribution	Commonly affecting telencephalon and diencephalon, brainstem less severely affected, cerebellum and spinal cord usually spared	Commonly affecting telencephalon, diencephalon and brainstem, cerebellum usually spared	Telencephalon and diencephalon, most severe in occipital and parietal lobes, tapering off rostral with the frontal lobes being less frequently affected, brainstem and cerebellum less frequently affected	Telencephalon and diencephalon, brainstem and cerebellum usually spared

White and gray matter involvement	In subcortical white matter of telencephalon with cortical gray matter being spared	Subcortical or deep white matter	May be dominating in the cerebral white matter or more commonly involve gray and white matter to a similar extend, loss of gray and white matter distinction	Cortical gray and white matter, sometimes a small rim of overlying cortical gray matter might be spared, deep white matter

Signal intensities	Hyperintense on T2 and FLAIR, mildly hypointense to isointense on T1, sometimes mildly hyperintense on T1	Hyperintense on T2 and FLAIR, hypointense to isointense on T1	Hyperintense on T2 and FLAIR, mildly hypo- to isointense on T1	Hyperintense on T2 and FLAIR, hypointense to isointense on T1

Midline shift	Possible to the more severely affected side	Not commonly seen, only mild if present	Common	Not commonly seen

Ventriculomegaly of lateral ventricles	Possible on the more severely affected side due to white matter loss	No	Can be seen	Can be seen, difficult to differentiate from normal ventriculomegaly in this breed

Brain herniation	No	No	Can be seen	No

Contrast enhancement	Mild to moderate, inhomogeneous or ring-like around a necrotic center, lesions with and without contrast enhancement may coexist in one patient, no meningeal enhancement	Varying, reaching from moderate to strong uniform, sometimes ring-like in the periphery of the lesion, no meningeal enhancement	Mild to moderate, inhomogeneous and patchy intraparenchymal pattern, meningeal enhancement is common, usually leptomeningeal enhancement	Mild intraparenchymal enhancement, meningeal enhancement not described

*NME, necrotizing meningoencephalitis; NLE, necrotizing leukoencephalitis; FLAIR, fluid-attenuated inversion recovery*.

## Future Developments

Despite the fact that imaging characteristics of NME and NLE have been outlined earlier, diagnosis is usually presumptive if based on imaging alone. Often, histological confirmation based on a brain biopsy is required to establish a definite diagnosis (20). However, performing brain biopsies is invasive and it is associated with certain morbidity. Therefore, non-invasive methods of imaging immunology are being investigated in human medicine. Inflammatory cells can be labeled with intravenously injected paramagnetic or superparamagnetic compounds (47, 48). Alternatively, cellular labeling can be achieved using other dipoles that are less common in normal tissue than hydrogen such as ^19^F, ^13^C, or ^15^N (47). Cells labeled this way may potentially be tracked by MRI in inflammatory and ischemic intracranial lesions allowing cellular characterization of encephalitis without the need for a histological examination.

## Author Contributions

TF has gathered all information and wrote the manuscript.

## Conflict of Interest Statement

The author declares that the research was conducted in the absence of any commercial or financial relationships that could be construed as a potential conflict of interest.

## Abbreviations

CSF, cerebrospinal fluid analysis; FDG-PET, fluorine-18 fluorodeoxyglucose; FLAIR, fluid-attenuated inversion recovery; GME, granulomatous meningoencephalomyelitis; MUE, meningoencephalitis of unknown etiology; NE, necrotizing encephalitis; NLE, necrotizing leucoencephalitis; NME, necrotizing meningoencephalitis; MR, magnetic resonance; MRI, magnetic resonance imaging; PET, positron emission tomography; SNE, subacute necrotizing encephalopathy.
